# Bubble-like lucency in pulmonary ground glass nodules on computed tomography: a specific pattern of air-containing space for diagnosing neoplastic lesions

**DOI:** 10.1186/s40644-024-00694-8

**Published:** 2024-04-02

**Authors:** Si-zhu Liu, Shi-hai Yang, Min Ye, Bin-jie Fu, Fa-jin Lv, Zhi-gang Chu

**Affiliations:** 1 Department of Radiology, The First Affiliated Hospital of Chongqing Medical University, 1# Youyi Road, Yuanjiagang, Yuzhong district, 400016 Chongqing, China; 2 Department of Radiology, People’s Hospital of Nanchuan district, 16# South street, Nanchuan district, 408400 Chongqing, China; 3 Department of Radiology, The First People’s Hospital of Neijiang, No.31 Tuozhong Road, Shizhong District, 641099 Neijiang, Sichuang Province China

**Keywords:** Lung neoplasms, Differential, Diagnosis, Vacuoles, Tomography, X-Ray computed

## Abstract

**Purpose:**

To investigate the computed tomography (CT) characteristics of air-containing space and its specific patterns in neoplastic and non-neoplastic ground glass nodules (GGNs) for clarifying their significance in differential diagnosis.

**Materials and methods:**

From January 2015 to October 2022, 1328 patients with 1,350 neoplastic GGNs and 462 patients with 465 non-neoplastic GGNs were retrospectively enrolled. Their clinical and CT data were analyzed and compared with emphasis on revealing the differences of air-containing space and its specific patterns (air bronchogram and bubble-like lucency [BLL]) between neoplastic and non-neoplastic GGNs and their significance in differentiating them.

**Results:**

Compared with patients with non-neoplastic GGNs, female was more common (*P* < 0.001) and lesions were larger (*P* < 0.001) in those with neoplastic ones. Air bronchogram (30.1% vs. 17.2%), and BLL (13.0% vs. 2.6%) were all more frequent in neoplastic GGNs than in non-neoplastic ones (each *P* < 0.001), and the BLL had the highest specificity (93.6%) in differentiation. Among neoplastic GGNs, the BLL was more frequently detected in the larger (14.9 ± 6.0 mm vs. 11.4 ± 4.9 mm, *P* < 0.001) and part-solid (15.3% vs. 10.7%, *P* = 0.011) ones, and its incidence significantly increased along with the invasiveness (9.5–18.0%, *P* = 0.001), whereas no significant correlation was observed between the occurrence of BLL and lesion size, attenuation, or invasiveness.

**Conclusion:**

The air containing space and its specific patterns are of great value in differentiating GGNs, while BLL is a more specific and independent sign of neoplasms.

**Supplementary Information:**

The online version contains supplementary material available at 10.1186/s40644-024-00694-8.

## Key points

**•** The air-containing space and its specific patterns (air bronchogram and BLL) were more common in neoplastic GGNs and can be seen as predictive indicators.
**•** The BLL can be seen as a specific sign of neoplastic GGNs, particularly in those with air bronchogram.
**•** In neoplastic GGNs, the occurrence of BLL is independent of lesion size, attenuation, and invasiveness.

**Supplementary Information:**

The online version contains supplementary material available at 10.1186/s40644-024-00694-8.

## Introduction

Lung cancer is a very common malignant tumor in the worldwide, which accounts for approximately 27% of all cancer deaths [1]. Early detection, diagnosis, and treatment are crucial for the better prognosis of lung cancers. In recent years, significant progress has been made in the treatment of non-small cell lung cancer (NSCLC), especially the immunotherapy, which has revolutionized the treatment landscape of NSCLC and represented a therapeutic breakthrough in this field [2, 3]. Synchronously, the factors that may affect the efficacy or modify the activity of immune checkpoint inhibitors (ICIs) in NSCLC patients were also studied and revealed, which further promoted the development of this therapy [2, 4]. Although the ICIs monotherapy is associated with improved survival, there is still controversy over whether chemoimmunotherapy or ICIs monotherapy is the first-line treatment for advanced NSCLC patients with high programmed cell death ligand 1(PD-L1) expression [5], which should be revealed in large-scale and well-designed clinical trials.

In recent years, low-dose computed tomography (CT) has become a widely used screening tool for lung cancer, proven to be effective in reducing associated mortality [6–8]. However, the increased utilization of CT has led to a significant rise in the detection of pulmonary ground-glass nodules (GGNs) [9]. These incidentally detected GGNs can be caused by various disorders, making them both neoplastic and non-neoplastic lesions, regardless of whether they are pure GGNs (pGGNs) or part-solid nodules (PSNs) [10–14]. Given the importance of differentiating neoplastic GGNs from non-neoplastic ones for clinical diagnosis and management, the differential diagnosis of GGNs has become a focus of radiological studies.

Radiological studies have identified significant differences in nodule size, density, boundary, and the presence of signs such as spiculation, lobulation, and vacuoles between neoplastic and non-neoplastic GGNs [15–18]. Non-neoplastic GGNs typically appear as pGGNs, while neoplastic GGNs often present as PSNs and exhibit signs of lobulation, vacuoles, and spiculation [16–22, 23]. However, these findings primarily provide evidence for possible diagnoses rather than specific diagnoses. Furthermore, there are ongoing debates about which characteristics hold greater value in the differential diagnosis. Consequently, the accurate diagnosis of GGNs is often hindered by the reliance on common CT features with varying incidences, necessitating further exploration into specific CT indicators for diagnosis.

The presence of air-containing spaces within GGNs, which is more commonly observed in neoplastic GGNs, has been frequently discussed as an indicator with significant value for differential diagnosis [18, 23–26]. However, the effectiveness of this sign in distinguishing GGNs has shown considerable variation across different studies, and some studies have even found it to be unreliable for differentiation [19, 27]. Currently, there is limited evidence from meta-analyses confirming the role of air-containing spaces in differentiating GGNs [28]. As a result, its practical application in clinical settings remains limited. After studying, two main reasons may be responsible for the variability of this sign in different studies. Firstly, there was a significant variation in sample sizes among the studies, with some studies having extremely small sample sizes that were not representative. Secondly, the air-containing space includes the vacuole sign or bubble-like lucency (BLL) and air bronchogram [18], which were often evaluated together or separately. However, the proportions of each of these patterns may differ in different samples, and there has been a lack of comparative analysis among them. Therefore, it is necessary to reevaluate the effectiveness of air-containing spaces and their specific patterns in differentiating GGNs and further explore their potential.

In this study, we aimed to investigate the CT characteristics of air-containing spaces and their specific patterns, as well as their performance in differentiating neoplastic and non-neoplastic GGNs. To achieve this, a larger sample of cases was enrolled and carefully evaluated. The results may provide more accurate and valuable information for the diagnosis and management of GGNs.

## Patients and methods

### Patients

This retrospective study was approved by the Institutional Review Board of the First Affiliated Hospital of Chongqing Medical University, and the requirement for informed consent was waived because of the retrospective nature of this study.

**Fig. 1:**
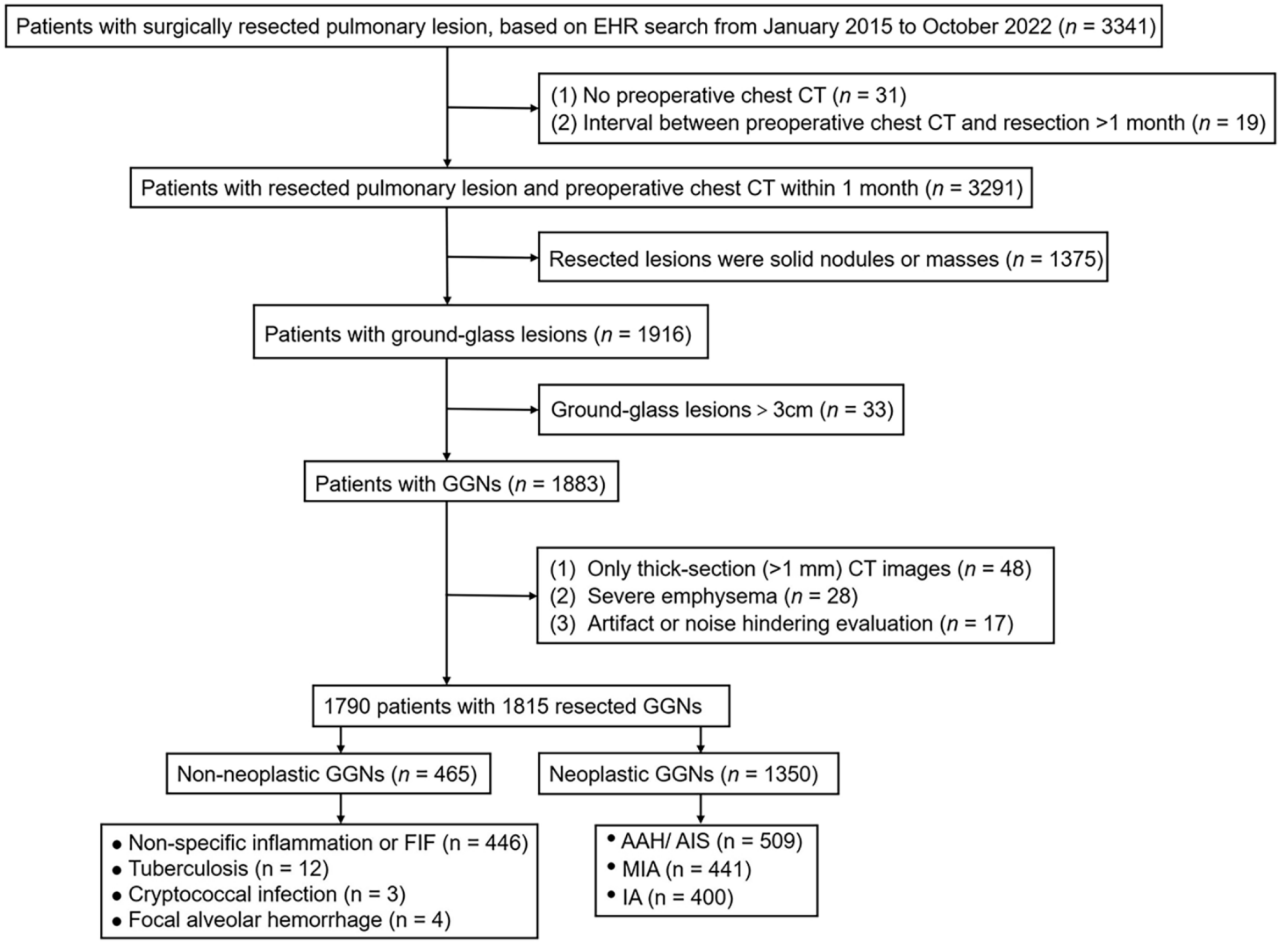
Flow chart of patient selection. GGN: ground-glass nodule; FIF = focal interstitial fibrosis; AAH: atypical adenomatous hyperplasia; AIS: adenocarcinoma in situ; MIA: minimally invasive adenocarcinoma; IAC: invasive adenocarcinoma

From January 2015 to October 2022, patients with surgically resected and pathologically confirmed neoplastic and non-neoplastic nodules at the same period were retrospectively enrolled in this study based on the electronic medical record system and picture archiving and communications system (PACS). Patients whose nodules were confirmed by lung biopsy or follow-up were not included because both trans-bronchial and trans-thoracic needle biopsy methods had limitations for pathologically confirming GGNs [29]. GGN is defined as a nodule that manifests as a ground-glass opacity (that does not completely obscure the underlying lung parenchyma within them) with or without any solid component (that obscures the underlying lung parenchyma other than blood vessels on lung window setting) on thin-section CT. The inclusion criteria were as follows: (1) nodules were GGNs; (2) lesions were smaller than 3 cm; and (3) patients’ clinical data were complete. The exclusion criteria were as follows: (1) CT images with the thickness > 1 mm; (2) the presence of artifacts on CT images affecting evaluation; and (3) the presence of emphysema on CT images because the blebs may mimic BLLs. Because the surgically resected GGNs are predominantly observed as nodules rather than masses, particularly in non-neoplastic cases, thus we did not include the lesions larger than 3 cm in our analysis. Finally, a total of 1790 patients, including 1328 patients with 1,350 neoplastic GGNs and 462 patients with 465 non-neoplastic GGNs, were enrolled in this study. Figure 1 shows the flow of patient selection. Among the 1790 patients, 1203 (67.2%) cases were found to have GGNs during routine screening for lung cancer, and 587 (32.8%) cases discovered GGNs incidentally during routine examinations for various other diseases, such as gastrointestinal conditions, cardiovascular issues, or emergency CT scans for chest pain.

### CT examinations

The patients were examined using one of the following CT scanners: SOMATOM Perspective (Siemens Healthineers, Erlangen, Germany), SOMATOM Definition Flash (Siemens Healthineers, Erlangen, Germany), and Discovery CT750 HD (GE Healthcare, Milwaukee, WI, USA). To minimize breathing artifacts, all CT scans were performed at the end of inspiration during a single breath-hold. The scan range was from the thoracic entrance to the costophrenic angle. The analysis was based on the unenhanced CT images, which were acquired with the following settings: tube voltage, 110−130 kVp; tube current, 50−140 mAs (reference mAs, using automatic current modulation technology); scanning slice thickness, 5 mm; rotation time, 0.5 s; pitch, 1−1.1; collimation, 0.6–0.625 mm; reconstruction slice thickness and interval, 0.625–1 mm; and matrix, 512 × 512. All images were reconstructed using a iterative reconstruction, and a standard algorithm (GE CT scanners) or medium-sharp algorithm (Siemens CT scanners) was adopted.

### Clinical data and image analysis

Based on the electronic medical record system and chest CT images, the patients’ clinical data were recorded. Clinical data, including the patient’s age, sex, smoking history, and concomitant diseases (pneumonia, bronchiectasis, tuberculosis, interstitial pneumonia, autoimmune diseases, cardiac disease) were collected.

The CT data were analyzed on a PACS workstation (Carestream Vue PACS) with lung window settings (window level, −600 HU; window width: 1500 HU). The multiple planner reconstruction images were used for detecting the air-containing space and determining their patterns. Two experienced radiologists specializing in chest imaging (Chu with 15 years of experience and Lv with 28 years of experience), who were blinded to the clinical data and histological diagnosis of the GGNs, independently assessed the CT scans. Any discrepancy between the two radiologists was resolved by consensus through fully displaying the characteristics of lesions or repeatedly measuring the parameters.

**Fig. 2:**
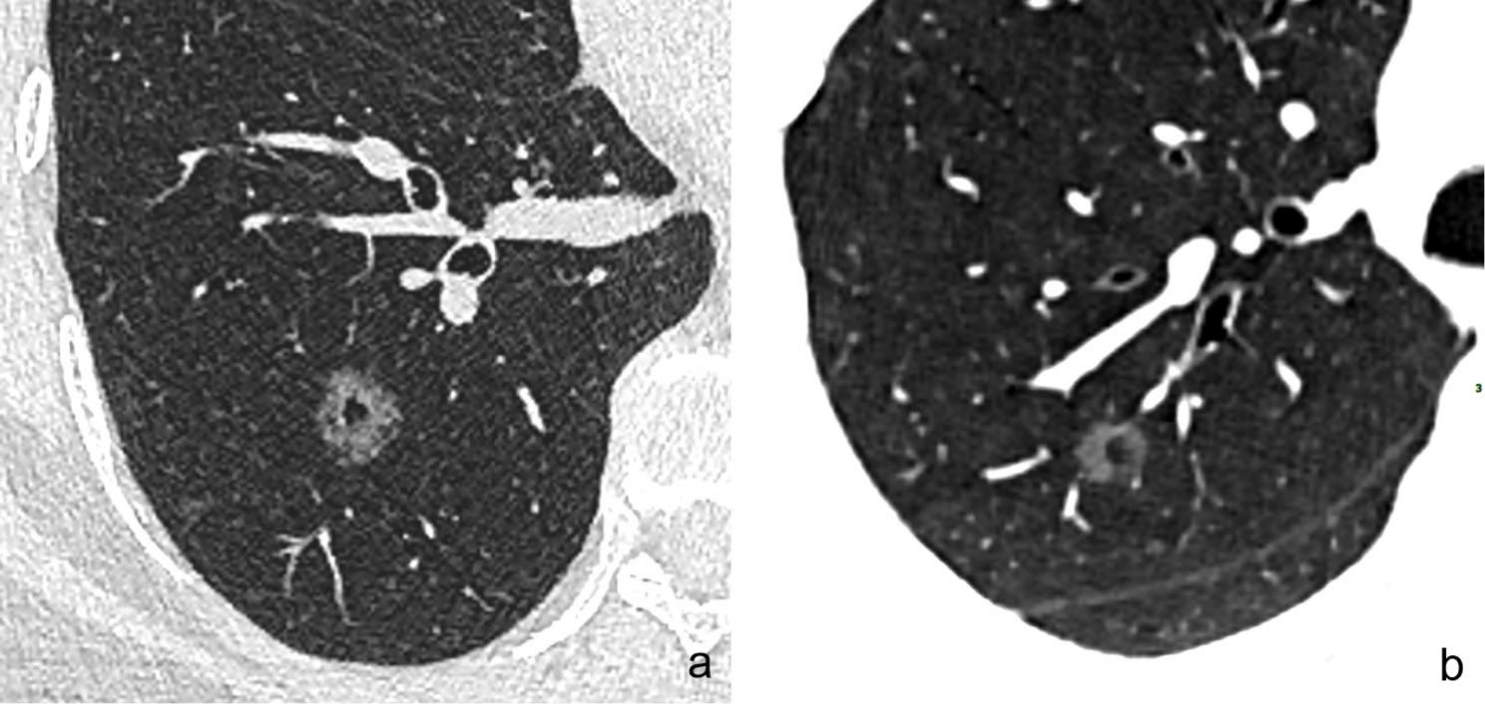
Different CT manifestations of the BLL sign in GGNs. (**a**) The density of BLL in the GGN (MIA) is lower than that of peripheral normal lung tissue and is similar to that of air attenuation. (**b**) The density of BLL in the GGN (AIS) is lower than that of peripheral tumor tissue, and which is similar to that of adjacent normal lung tissue. BLL: bubble-like lucency; GGNs: ground glass nodules; MIA: minimally invasive adenocarcinoma; AIS: adenocarcinoma in situ

**Fig. 3:**
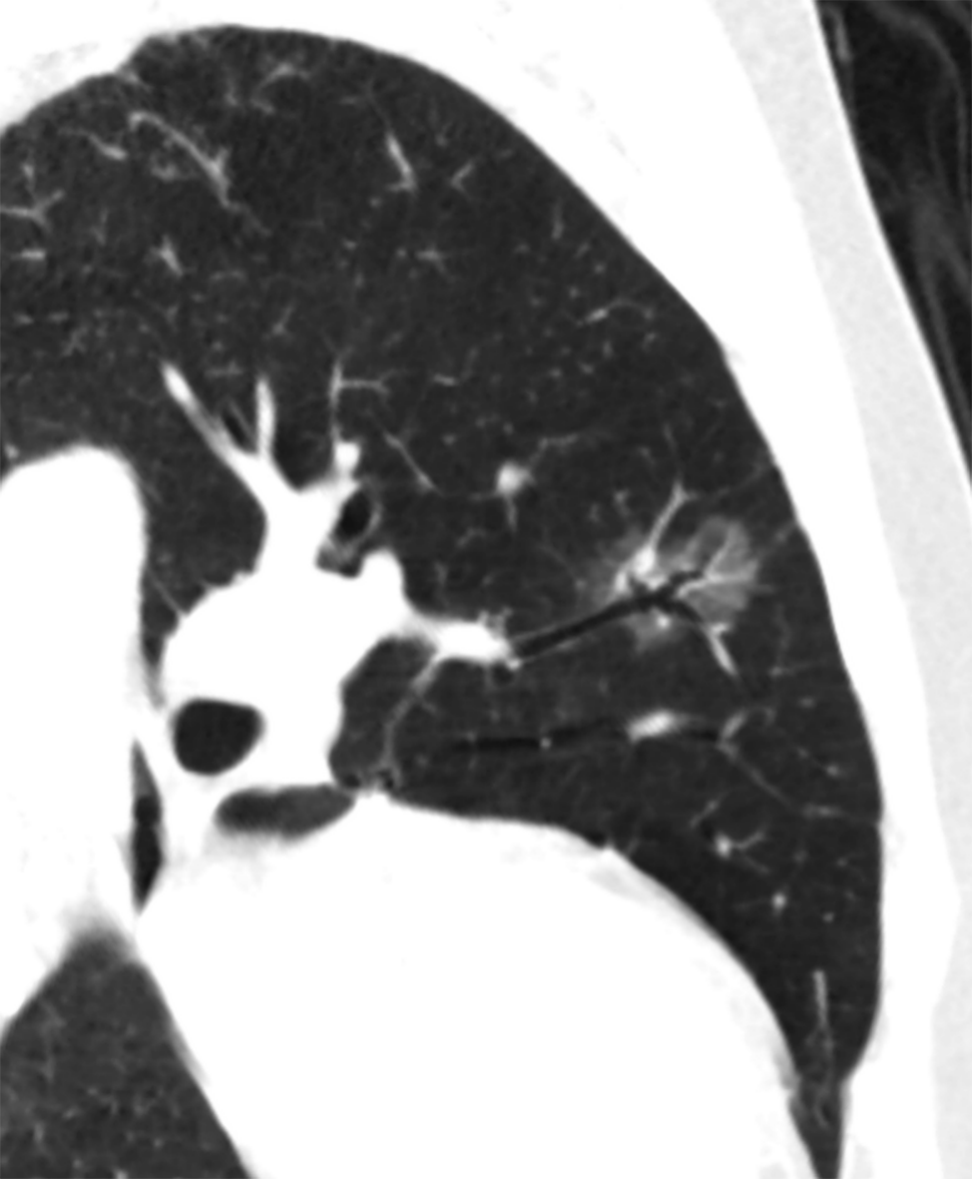
The air bronchogram sign in GGN (IAC) on multiplanar reformation image, which is manifested as branching air density connecting with proximal bronchus. GGN: ground glass nodule. IAC: invasive adenocarcinoma

**Fig. 4:**
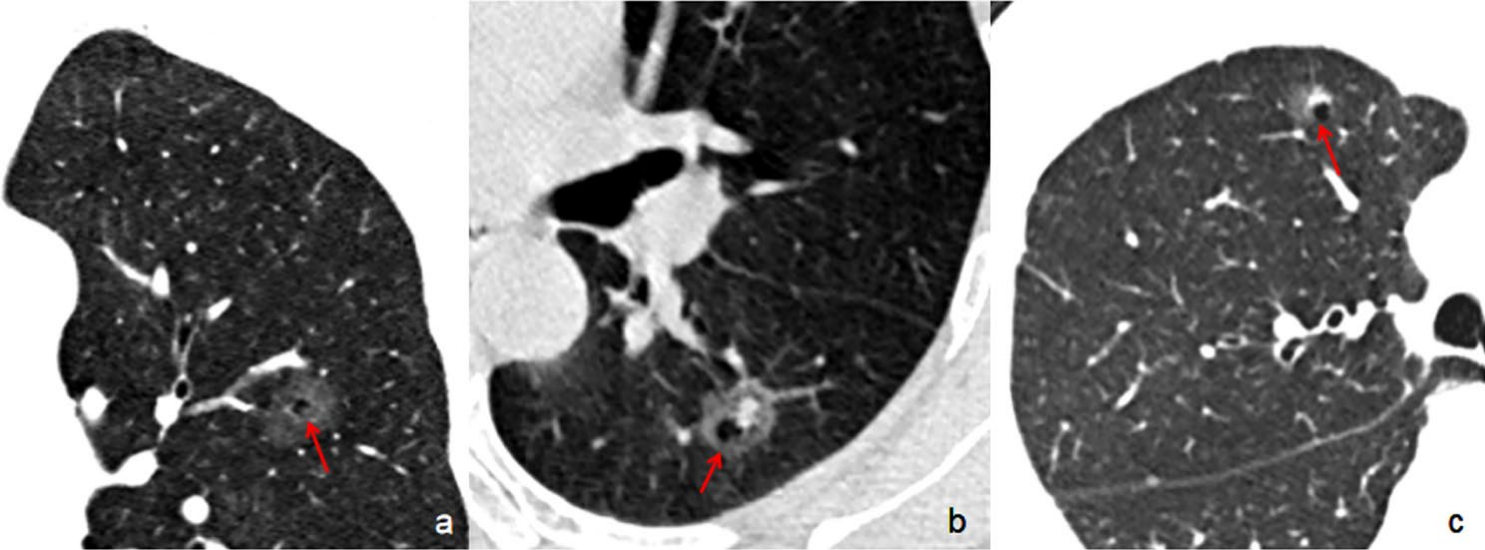
The different locations of BLL in GGNs. (**a**) Central type: the BLL locates in the center of the nodule (AAH), (**b**) peripheral type: the BLL locates in the nodule (MIA) but not in the center. (**c**) Marginal type: the BLL locates in the junction between the nodule (MIA) and the peripheral normal lung. BLL: bubble-like lucency; GGNs: ground glass nodules. AAH: atypical adenomatous hyperplasia; MIA: minimally invasive adenocarcinoma

The following CT features of GGNs were analyzed: (a) nodule size (the mean of the longest diameter and the perpendicular diameter on axial CT images), (b) location (upper lobe, middle lobe, or lower lobe), (c) attenuation on CT images (pGGN or PSN), (d) shape (regular [round or oval] or irregular), (e) boundary (well-defined or ill-defined), (f) lobulation sign (yes or no), (g) spiculation (yes or no), (h) pleural indentation (yes or no), (i) air-containing space, (j) air bronchogram, and (k) BLL: number, size, shape, and location. Based on the presence/absence of a solid component within the nodule on CT images in the lung window, we classified the nodules as PSN or pGGN [30]. An air-containing space included BLL and air bronchogram. The BLL sign was defined as small spots of air attenuation or low density similar to normal lung tissue within the lesion [31] (Fig. 2), and the air bronchogram sign was defined as visible branching or tubular air density within a nodule due to the reduced air content of the surrounding tissues [18, 32] (Fig. 3). According to the location of BLL in GGNs, it was classified as central type (located in the center of the nodule), peripheral type (located in the nodule but not in the center), and marginal type (located in the junction between the nodule and the peripheral normal lung) (Fig. 4a–c). Air bronchograms were divided into two types: natural (without dilation or distortion) and dilated (the lumen diameter of the part in the nodule is greater than or equal to that of the proximal part outside the nodule) or distorted.

### Statistical analysis

The patients’ clinical data and CT features of air-containing space were statistically analyzed using Statistical Package for the Social Sciences (version 25.0; IBM Corp., Armonk, NY, USA). Continuous variables were expressed as means ± standard deviations, and categorical variables were expressed as numbers and percentages. For comparing differences in variables between neoplastic and non-neoplastic GGNs, the patients’ age was analyzed using Student’s t-test; the Mann–Whitney U-test was used for nodule size, and the Pearson chi-square test was used for gender, smokers, concomitant diseases; attenuation and distribution of nodules; CT features of nodules (shape, boundary, spiculation, lobulation, and pleural indentation); the incidence of air-containing space, air bronchogram, and BLL; the incidence of single and multiple patterns of air containing space; and the incidence of BLL in neoplastic GGNs with different pathological types and CT patterns. Pearson chi-square test was also used for comparing the incidences of air bronchogram in GGNs with BLL of different shapes. Baseline CT variables with statistical difference in univariate analysis were further included in the binary logistic regression analysis with the aim of determining whether they were independent factors for predicting neoplastic GGNs. Besides, Pearson correlation analysis was performed to analyze the relationship between the occurrence of BLL and the size, CT pattern, and pathological type of neoplastic GGNs. A receiver operating characteristic (ROC) curve analysis was performed to evaluate the diagnostic value of the air containing space, air bronchogram, BLL, and other morphological feature for predicting whether GGNs were neoplastic or non-neoplastic. Differences with *p*-values of less than 0.05 were considered statistically significant.

## Results

### Patients’ clinical characteristics and CT features of GGNs

Table 1 summarizes the baseline characteristics of the patients and the detected GGNs on CT images. The proportion of females was significantly higher in patients with neoplastic GGNs than in those with non-neoplastic GGNs (*P* < 0.001). Neoplastic GGNs were significantly larger than the non-neoplastic ones (*P* < 0.001). The percentages of non-neoplastic GGNs located in the inferior lobes and upper lobes were higher (37.4% vs. 26.9%, *P* < 0.001) and lower (56.6% vs. 66.6%, *P* < 0.001) than those of neoplastic GGNs, respectively.

**Table 1:** Baseline characteristics of patients and GGNs

Characteristics	Patients with non-neoplastic GGNs (n = 462)	Patients with neoplastic GGNs (n = 1328)	*P*-value
Mean age (year) (range)	53.8 ± 11.3 (16 ~ 78)	55.0 ± 11.4 (22 ~ 88)	0.041
Gender (Male / Female)	214 (46.3) /248 (53.7)	422 (31.8) /906 (68.2)	< 0.001
Smokers	95 (20.6)	303 (22.8)	0.316
Patients with concomitant diseases			
Pneumonia	84 (18.2)	278 (20.9)	0.205
Bronchiectasis	37 (8.0)	92 (6.9)	0.439
Tuberculosis	7 (1.5)	16 (1.2)	0.601
Interstitial pneumonia	10 (2.2)	43 (3.2)	0.241
Autoimmune diseases	14 (3.0)	28 (2.1)	0.260
Cardiac disease	12 (2.6)	59 (4.4)	0.080
GGNs	465	1350	
Size (mm) (range)	10.1 ± 4.5 (4 ~ 29)	11.9 ± 5.2 (4 ~ 29)	< 0.001
Location			
Right upper lobe	159 (34.2)	536 (39.7)	< 0.001
Right middle lobe	28 (6.0)	88 (6.5)	
Right inferior lobe	105 (22.6)	225 (16.7)	
Left upper lobe	104 (22.4)	363 (26.9)	
Left inferior lobe	69 (14.8)	138 (10.2)	
Attenuation on CT images			
Pure GGN	190 (41.1)	684 (50.7)	< 0.001
PSN	274 (58.9)	666 (49.3)	
Pathological nature			
AAH	/	95 (7.0)	
AIS	/	414 (30.7)	
MIA	/	441 (32.7)	
IAC	/	400 (29.6)	
Non-specific inflammation or FIF	446 (95.9)	/	
Tuberculosis	12 (2.6)	/	
Cryptococcal infection	3 0.6)	/	
Focal alveolar hemorrhage	4 (0.9)	/	
Surgical procedure			< 0.001
Wedge resection	138 (29.7)	304 (22.5)	
Segmentectomy	255 (54.8)	581 (43.0)	
Lobectomy	72 (15.5)	465 (34.5)	

*Note* Data are expressed as mean ± SD or n (%). GGN = ground glass nodule, PSN = part solid nodule, AAH = atypical adenomatous hyperplasia, AIS = adenocarcinoma in situ, MIA = minimally invasive adenocarcinoma, IAC = invasive adenocarcinoma, FIF = focal interstitial fibrosis

### Binary logistic regression analysis of the CT characteristics of nodules

Table 2 shows the CT characteristics that independently discriminated neoplastic GGNs from non-neoplastic ones via binary logistic regression analysis. Compared with non-neoplastic GGNs, the air bronchogram, BLL, lesion size, regular shape, and well-defined boundary were found to be the significantly independent indicators of neoplastic ones.

**Table 2:** Binary logistic regression analysis of the CT characteristics of nodules

CT features	Non-neoplastic GGNs (n = 465)	Neoplastic GGNs (n = 1350)	*P*-value	OR	95% CI
Air bronchogram	80 (17.2)	407 (30.1)	0.013	1.534	1.095–2.149
BLL	12 (2.6)	175 (13.0)	< 0.001	4.245	2.259–7.978
Size (mm)	10.1 ± 4.5 (4 ~ 29)	11.9 ± 5.2 (4 ~ 29)	< 0.001	1.157	1.114–1.202
Regular shape	291 (62.6)	1117 (82.7)	< 0.001	7.287	5.164–10.281
Well-defined boundary	233 (50.1)	1114 (82.5)	< 0.001	4.211	3.253–5.452
Lobulation sign	87 (18.7)	451 (33.4)	0.916	0.983	0.710–1.361
Pleural indentation	51 (11.0)	214 (15.9)	0.361	1.208	0.805–1.814

*Note* Data are expressed as n (%). BLL: bubble-like lucency

*Abbreviations* CI: confidence interval; OR: odds ratio

### Comparison of the diagnostic performance of air-containing space and other CT features of nodules

Table 3 shows the air-containing space and its specific patterns as well as other common CT features in neoplastic and non-neoplastic GGNs. Air bronchogram (30.1% vs. 17.2%), BLL (13.0% vs. 2.6%), regular shape (82.7% vs. 62.6%), well-defined boundary (82.5% vs. 50.1%), lobulation sign (33.4% vs. 18.7%), and pleural indentation (15.9% vs. 11.0%) were more commonly detected in neoplastic GGNs than in non-neoplastic GGNs (each *P* < 0.05). Comparing the abilities of the different patterns of air-containing space and other morphological features in diagnosing neoplastic GGNs, the former ones had relatively lower sensitivity but higher specificity than the latter ones, and the BLL had the highest specificity (93.6%).

**Table 3:** Performance of air-containing space and other CT features in differentiating GGNs

CT features	Non-neoplastic GGNs (n = 465)	Neoplastic GGNs (n = 1350)	*P*-value	ROC analysis			
				AUC (95% CI)	Sensitivity (%)	Specificity (%)	*P*-value
Air bronchogram	80 (17.2)	407 (30.1)	< 0.001	0.565 (0.536–0.594)	30.1	82.8	< 0.001
BLL	12 (2.6)	175 (13.0)	< 0.001	0.607 (0.570–0.644)	27.8	93.6	< 0.001
Regular shape	291 (62.6)	1117 (82.7)	< 0.001	0.601 (0.570–0.632)	82.7	37.4	< 0.001
Well-defined boundary	233 (50.1)	1114 (82.5)	< 0.001	0.661 (0.631–0.692)	82.7	49.6	< 0.001
Spiculation sign	35 (7.5)	115 (8.5)	0.287	0.512 (0.465–0.560)	23.3	74.2	0.613
Lobulation sign	87 (18.7)	451 (33.4)	< 0.001	0.567 (0.539–0.595)	16.2	70.4	0.014
Pleural indentation	51 (11.0)	214 (15.9)	0.005	0.537 (0.501–0.574)	19.2	73.3	0.052

*Note* Data are expressed as n (%). BLL: bubble-like lucency

### Comparison of air bronchogram and BLL in neoplastic and non-neoplastic GGNs

**Fig. 5:**
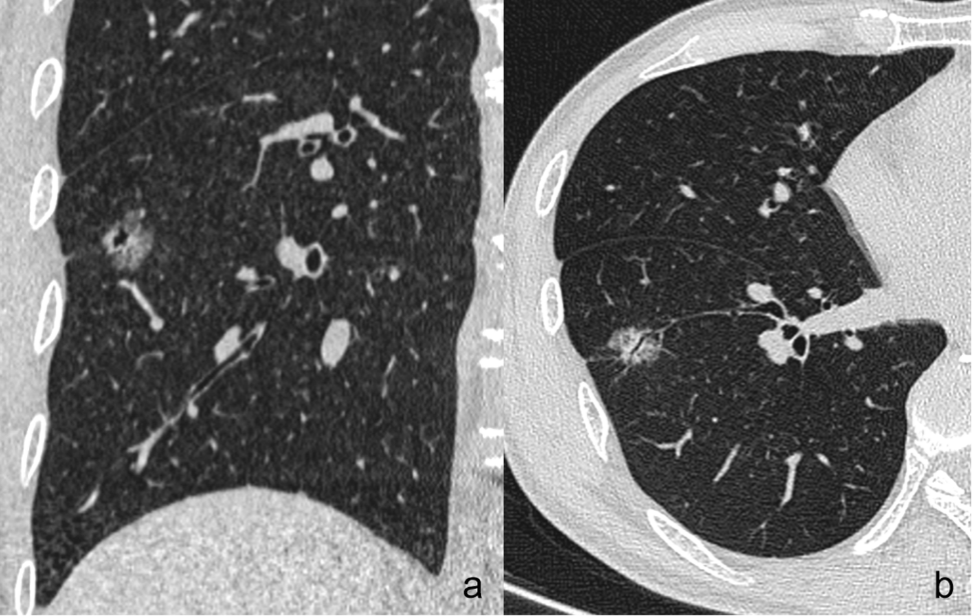
A 49-year-old male has a invasive adenocarcinoma located in the right lower lobe. (**a**) Coronal image shows there is an irregular peripheral type BLL in the GGN. (**b**) Axial image shows the concomitant air bronchogram sign at another section. BLL: bubble-like lucency; GGN: ground glass nodule

**Fig. 6:**
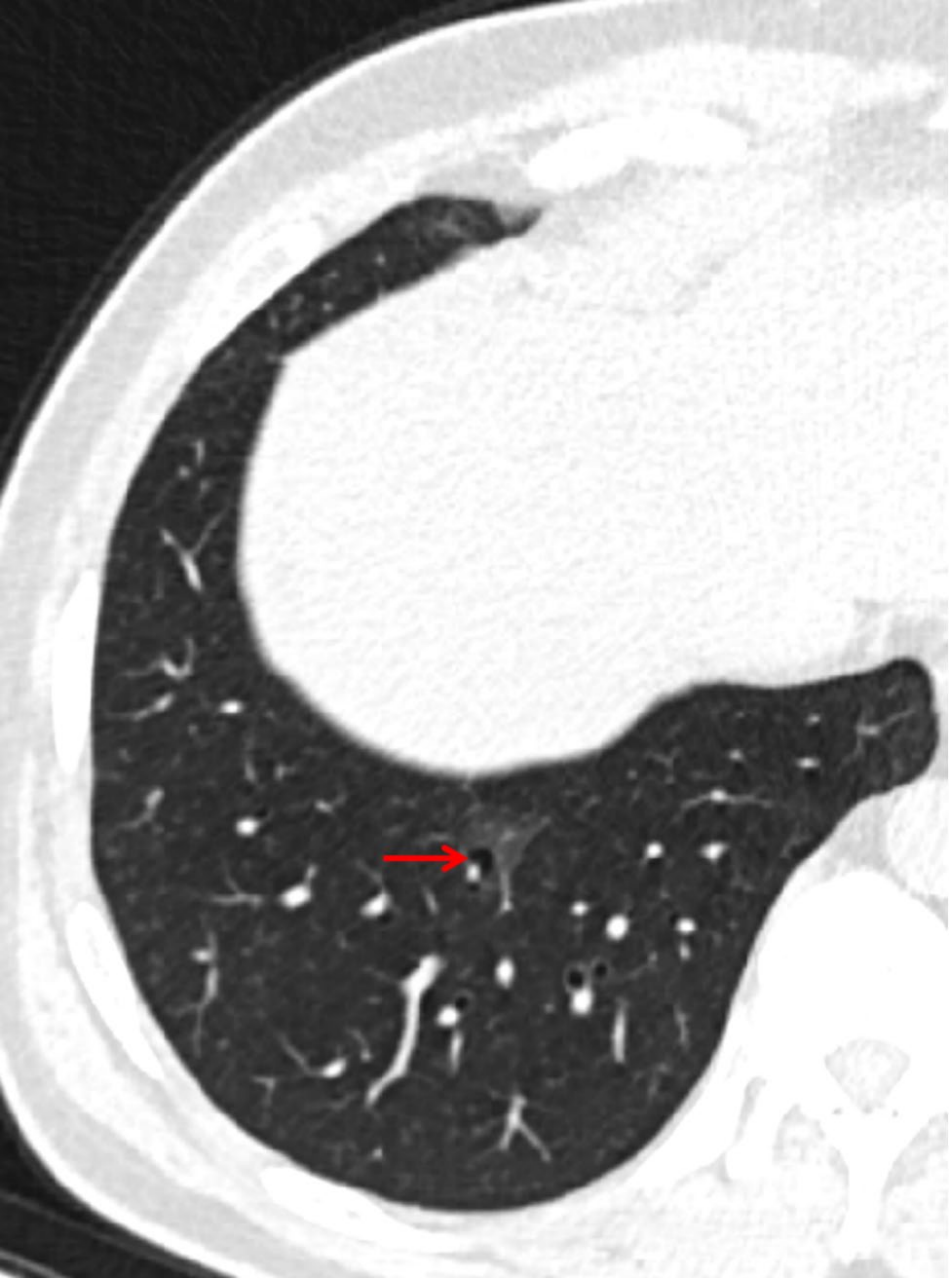
A 52-year-old male has a persisted GGN with a marginal type BLL located in the right lower lobe. It is confirmed as focal interstitial fibrosis after operation. GGN: ground glass nodule; BLL: bubble-like lucency

Among the GGNs with air bronchogram, the incidence rates of dilated or distorted bronchi in neoplastic (*n* = 109, 26.8%) and non-neoplastic (*n* = 25, 31.3%) GGNs were similar (*P* = 0.413). The CT characteristics of BLL in neoplastic and non-neoplastic GGNs are shown in Table 4. The numbers of BLLs that were oval or irregular (*P* = 0.005) and located in the central or peripheral area (*P* < 0.001) were all greater in neoplastic GGNs than those in non-neoplastic GGNs (Fig. 5). Among the BLLs in 12 non-neoplastic GGNs, more than half were marginal type (Fig. 6).

**Table 4:** CT characteristics of the BLL sign in neoplastic and non-neoplastic GGNs

Characteristics	Non-neoplastic GGNs (n = 12)	Neoplastic GGNs (n = 175)	*P*-value
Size (mm) (range)	3.07 ± 0.82 (1.7–4.0)	4.54 ± 2.15 (1.5–15)	0.019
Single/multiple	10/2	129/46	0.734
Density			0.528
Air density	11 (91.7)	165 (94.3)	
Similar to normal lung tissue	1 (8.3)	10 (5.7)	
Shape			0.005
Round	5 (41.7)	22 (12.6)	0.019
Oval	7 (58.3)	97 (55.4)	0.845
Irregular	0	56 (32)	0.019
Location in nodule			< 0.001
Central type	1 (8.3)	45 (25.7)	0.314
Peripheral type	4 (33.4)	124 (70.9)	0.017
Marginal type	7 (58.3)	3 (1.7)	< 0.001
Peripheral and marginal type	0	3 (1.7)	1.000
Concomitant air bronchogram	1(8.3)	65 (37.4)	0.088
GGNs with round BLL	1(8.3)	7 (4.0)	0.379
GGNs with oval BLL	/	41 (23.4)	
GGNs with irregular BLL	/	17 (9.7)	

*Note* Data are expressed as mean ± SD or n (%). GGNs = ground glass nodules

### Characteristics of BLL in neoplastic GGNs

The neoplastic GGNs with BLL were significantly larger than those without BLL (14.9 ± 6.0 vs. 11.4 ± 4.9, *P* < 0.001). Among the 175 neoplastic GGNs with BLL, 73 were pGGNs and 102 were PSNs, respectively. Its incidence was higher in PSNs than in pGGNs (15.3% vs. 10.7%, *P* = 0.011). Among the atypical adenomatous hyperplasia (AAH), adenocarcinoma in situ (AIS), minimally invasive adenocarcinoma (MIA), and invasive adenocarcinoma (IAC), the number of lesions with BLL was 9 (9.5%), 36 (8.7%), 58 (13.2%), and 72 (18.0%), respectively, which significantly increased along with the invasiveness of GGNs (*P* = 0.001). Correlation analysis suggested that the occurrence of BLL was not closely correlated with the tumor size (*r* = 0.226; *P* < 0.001), attenuation on CT images (*r* = 0.069; *P* = 0.011), and degree of invasiveness (*r* = 0.106; *P* < 0.001).

## Discussion

In this study, the performance of air-containing space and its specific patterns in differentiating neoplastic GGNs from non-neoplastic ones was compared based on a larger sample. It was found that the air-containing space, air bronchogram, and BLL signs were significantly more common in neoplastic GGNs than in non-neoplastic ones. Therefore, each of these signs can be considered as an indicator of neoplastic GGNs. Among these signs, the BLL sign demonstrated the highest specificity (93.6%) among the common morphological features. Interestingly, in neoplastic GGNs, the occurrence of BLL was not closely correlated with tumor size, attenuation on CT images, or the degree of invasiveness. Overall, any pattern of air-containing space is valuable in differentiating GGNs, and the BLL sign can be considered as a specific and independent indicator for neoplastic GGNs. These findings provide precise information for the diagnosis and management of GGNs.

The air bronchogram sign was frequently observed in both neoplastic and non-neoplastic GGNs, probably reflecting the preservation of the underlying pulmonary architecture. The difference in the prevalence is probably because most non-neoplastic GGNs are infiltrative and therefore opacify the pulmonary airspaces more easily; however, in neoplastic GGNs, cellular infiltrates were less common [18]. Furthermore, there was no significant difference in the incidence of natural or dilated and distorted bronchi between neoplastic and non-neoplastic GGNs [23]. The present study confirmed these previous findings, suggesting that the presence of the air bronchogram sign and changes in the internal bronchus cannot be considered specific predictors of neoplastic GGNs.

In contrast, the presence of the BLL sign was predominantly observed in neoplastic GGNs, which can be attributed to the lepidic tumor growth pattern that leads to incomplete filling of the normal parenchyma, dilated bronchium, and enlarged alveolar spaces [22]. When compared to the air bronchogram sign, although the BLL sign was less common in neoplastic GGNs, it may be a more specific indicator, supporting previous speculation based on smaller sample size [18]. However, it is worth noting that the BLL sign was not exclusively detected in tumors. Some non-neoplastic GGNs also exhibited the BLL sign, but more than a half cases were of the marginal type, which is rarely observed in neoplastic GGNs. Additionally, almost all non-neoplastic GGNs with the BLL sign did not show concomitant air bronchogram. Therefore, the presence of a marginal BLL sign may indicate a non-neoplastic GGN, while the coexistence of the BLL and air bronchogram signs strongly suggests a tumor. These findings were not reported previously and should be verified in the future studies.

In this study, the differences in the prevalence of air bronchogram and BLL sign in neoplastic and non-neoplastic GGNs are consistent with previous research [18, 19, 23, 26]. However, there are some studies that have reported different findings. For example, one study that included 94 non-neoplastic GGNs and 1,840 neoplastic GGNs found that BLL and air bronchogram signs were more common in neoplastic GGNs. However, after adjusting for baseline characteristics, only the BLL sign was found to be more common in matched neoplastic PSNs [19]. Another study involving 33 non-neoplastic GGNs and 47 neoplastic GGNs found that only the air bronchogram sign was more common in neoplastic pGGNs. The incidence rates of the BLL sign in non-neoplastic and neoplastic GGNs were similar, regardless of whether they were pure or mixed [11]. Furthermore, both the BLL and air bronchogram signs were rarely detected in transient pure GGNs [33]. These differences in findings may be attributed to variations in study methods or relatively small sample sizes.

Among neoplastic GGNs, the BLL sign was more frequently observed in large, part-solid, and highly aggressive tumors. However, its occurrence did not correlate with tumor size, CT attenuation, or invasiveness. Similar findings have been reported in previous studies, where the occurrence rate of the BLL sign in invasive adenocarcinoma and pre-invasive lesions showed no significant difference, regardless of whether they were pGGNs or PSNs [34, 35]. Therefore, the diagnostic value of the BLL sign in distinguishing between pre-invasive and invasive GGNs is limited [15, 36]. It can be concluded that the occurrence of the BLL sign in tumors is highly random, and this sign serves as an independent indicator with significant value only in differentiating GGNs.

Among neoplastic GGNs, the BLL sign was more commonly detected in the large, part-solid, and highly aggressive ones. However, its occurrence did not correlate with the tumor size, attenuation on CT images, or degree of invasiveness. Similar findings have been reported in previous studies, where the occurrence rate of the BLL sign in invasive adenocarcinoma and pre-invasive lesions showed no significant difference, regardless of whether they were pGGNs or PSNs [34, 35]. Therefore, the diagnostic value of the BLL sign in distinguishing between pre-invasive and invasive GGNs is limited [15, 36]. It can be concluded that the occurrence of the BLL sign in tumors is highly random, and this sign serves as an independent indicator with significant value only in differentiating GGNs.

Though the air bronchogram and BLL signs are valuable indicators for distinguishing GGNs, they are not commonly observed, especially the BLL sign. Therefore, it is important to consider other morphological characteristics in the diagnosis of GGNs. A study has suggested that factors such as nodule size, CT attenuation, lesion border, and margin type in solitary GGNs can be useful in differentiating lung cancer from benign lesions. It has been confirmed that the presence of a well-defined border and a higher average CT value are associated with malignancy in pGGNs, and the presence of a larger size, well-defined border, and spiculated margin can aid in the differential diagnosis of malignant PSNs [19]. However, in this study, any type of air-containing space, particularly the BLL sign, showed significantly higher specificity compared to other morphological features in diagnosing neoplastic GGNs. Regarding the smaller GGNs without the aforementioned indicators, the air containing space, BLL in particular, could be considered an important clue for discriminating them.

Note that the “BLL sign” rather than the “vacuole sign” was used for describing localized low-density area in GGNs in this study because the latter is typically defined as round or irregular air attenuation with a diameter of 1–2 mm in a nodule [22]. However, in the cases included in this study, the diameter of the low-density areas reached up to 15 mm. Despite the size difference, we believe that the nature of the low-density areas in GGNs is similar, regardless of their size. Furthermore, it is important to exercise caution when interpreting the presence of the BLL sign in patients with emphysema. In these cases, the presence of the BLL sign may be attributed to pre-existing internal bullae surrounded by the lesions, leading to a potentially “false” BLL sign. Therefore, when distinguishing GGNs, particularly in patients with emphysema, the presence of the BLL sign should be interpreted with caution.

GGNs are a common type of pulmonary disease that can exhibit various characteristics and frequently require differential diagnosis. However, compared to solid nodules, GGNs have fewer CT features such as lobulation, spiculation, and pleural indentation, which makes their differential diagnosis more challenging. Therefore, there is a need for more specific CT features to improve differentiation. Previous and the present studies have shown that air containing space, including air bronchogram and BLL, is more frequently observed in neoplastic lesions. However, there has been no research on their specificity in diagnosis, leading to the neglect of their value in differential diagnosis. In the present study, based on a large sample, it was found that although the sensitivity of BLL was not high, its specificity reached 93.6%. This finding confirms its importance in differential diagnosis, especially for the inexperienced radiologists, as it improves their diagnostic accuracy of GGNs to some certain extent. The future trend in imaging diagnosis is precise diagnosis, which requires the exploration of more specific imaging features for accurate differentiation. Additionally, incorporating more specific features discovered in clinical studies into artificial intelligence software for lung nodule assessment may potentially enhance its ability to predict the neoplastic or non-neoplastic nature of GGNs in the future.

### Limitations

This study has several limitations that should be acknowledged. Firstly, it is important to note that this was a retrospective study conducted on a larger sample size. Therefore, it is crucial to validate these findings through prospective studies or in a real clinical setting. Secondly, there may be patient selection bias in this study as it only included surgically treated GGNs with pathological results. This means that the conditions of air-containing space in non-surgically treated GGNs, or those that resolve on their own, are unknown. Thirdly, while this study evaluated the performance of the air bronchogram and BLL sign in differentiating GGNs, it did not provide a comprehensive evaluation of other morphological features that have been investigated in prior studies. Finally, given the low sensitivity of the BLL sign, it is recommended to combine it with other clinical and radiological features such as lobulation, spiculation, and pleural indentation signs to accurately assess the possibility of GGNs in clinical practice.

## Conclusions

In conclusion, by investigating the performance of air-containing space and its specific patterns (air bronchogram and BLL) in differentiating GGNs based on a larger sample in patients without emphysema, it was found that any one of them was more common in neoplastic GGNs and can be seen as predictive indicators. Compared with air bronchogram, the BLL sign was less common but had higher specificity in diagnosing neoplastic GGNs. Once a GGN with the BLL sign is found, particularly that with concomitant air bronchogram, a diagnosis of a neoplastic lesion can almost be made. However, if only a marginal type BLL is detected, the possibility of a non-neoplastic GGN should be considered, and follow-up is recommended as the first step.

### Electronic supplementary material

Below is the link to the electronic supplementary material.

Supplementary Material 1

## Data Availability

The datasets used and/or analyzed during the current study are available from the corresponding author on reasonable request.

## Abbreviations

CT: computed tomography
GGNs: ground glass nodules
BLL: bubble-like lucency
pGGNs: pure GGNs
PSNs: part-solid nodules
AAH: atypical adenomatous hyperplasia
AIS: adenocarcinoma in situ
MIA: minimally invasive adenocarcinoma
IAC: invasive adenocarcinoma
