# Supplementary material for: Bubble-like lucency in pulmonary ground glass nodules on computed tomography: a specific pattern of air-containing space for diagnosing neoplastic lesions
Source: Cancer Imaging. 2024 Apr 2;24:47. doi: 10.1186/s40644-024-00694-8 (PMC10985942; doi:10.1186/s40644-024-00694-8)
Supplement: Supplementary file 1 — Supplementary Material 1 [file 40644_2024_694_MOESM1_ESM.docx]

Dear Editor:

Here we submit our **revised** research article entitled “**Bubble-like lucency in pulmonary ground glass nodules on computed tomography: a specific pattern of air-containing space for diagnosing neoplastic lesions (CAIG-D-23-00694)**” to the editorial office of **Cancer Imaging**.

On behalf of my co-authors, we thank you so much for your letter and giving us an opportunity to resubmit our manuscript. These suggestions are very helpful for revising and improving our paper, as well as the important guiding significance to other researches. We have studied the comments carefully and made corresponding corrections which we hope meet with approval. The main corrections are in the revised manuscript and the responds to the reviewers’ comments are as follows (the replies are highlighted in blue).

Replies to the reviewers’ comments:

Reviewer #1:

**Q:** Introduction section: although the authors correctly included important papers in this setting, we believe the evolving systemic treatment scenario for lung cancer should be briefly discussed and some recently published papers added within the introduction (PMID: 35326555; PMID: 35031442; PMID: 36064585; PMID: 37535194), only for a matter of consistency.

**Reply:** Thanks. Based on your suggestion, we have read these articles and added some relevant contents in the introduction section.

**Changes in the text:**

Lung cancer is a very common malignant tumor in the worldwide, which accounts for approximately 27% of all cancer deaths [1]. Early detection, diagnosis, and treatment are crucial for the better prognosis of lung cancers. In recent years, significant progress has been made in the treatment of non-small cell lung cancer (NSCLC), especially the immunotherapy, which has revolutionized the treatment landscape of NSCLC and represented a therapeutic breakthrough in this field [2, 5]. Synchronously, the factors that may affect the efficacy or modify the activity of immune checkpoint inhibitors (ICIs) in NSCLC patients were also studied and revealed, which further promoted the development of this therapy [2, 3]. Although the ICIs monotherapy is associated with improved survival, there is still controversy over whether chemoimmunotherapy or ICIs monotherapy is the first-line treatment for advanced NSCLC patients with high programmed cell death ligand 1(PD-L1) expression [4], which should be revealed in large-scale and well-designed clinical trials.

**Q:** Discussion section: Of note, the authors should expand the Discussion section, including a more personal perspective to reflect on. For example, they could answer the following questions - in order to facilitate the understanding of this complex topic to readers: what potential does this study hold? What are the knowledge gaps and how do researchers tackle them? How do you see this area unfolding in the next 5 years?

**Reply:** Thanks. Based on your suggestion, we have added some relevant contents in the discussion section.

**Changes in the text**:

GGNs are a common type of pulmonary disease that can exhibit various characteristics and frequently require differential diagnosis. However, compared to solid nodules, GGNs have fewer CT features such as lobulation, spiculation, and pleural indentation, which makes their differential diagnosis more challenging. Therefore, there is a need for more specific CT features to improve differentiation. Previous and the present studies have shown that air containing space, including air bronchogram and BLL, is more frequently observed in neoplastic lesions. However, there has been no research on their specificity in diagnosis, leading to the neglect of their value in differential diagnosis. In the present study, based on a large sample, it was found that although the sensitivity of BLL was not high, its specificity reached 93.6%. This finding confirms its importance in differential diagnosis, especially for the inexperienced radiologists, as it improves their diagnostic accuracy of GGNs to some certain extent. The future trend in imaging diagnosis is precise diagnosis, which requires the exploration of more specific imaging features for accurate differentiation. Additionally, incorporating more specific features discovered in clinical studies into artificial intelligence software for lung nodule assessment may potentially enhance its ability to predict the neoplastic or non-neoplastic nature of GGNs in the future.

**Q:** One additional little flaw: the authors could better explain the limitations of their work, in the last part of the Discussion.

**Reply:** Thanks for your suggestion. We have enriched the limitations of the article.

**Changes in the text**: This study has several limitations that should be acknowledged. Firstly, it is important to note that this was a retrospective study conducted on a larger sample size. Therefore, it is crucial to validate these findings through prospective studies or in a real clinical setting. Secondly, there may be patient selection bias in this study as it only included surgically treated GGNs with pathological results. This means that the conditions of air-containing space in non-surgically treated GGNs, or those that resolve on their own, are unknown. Thirdly, while this study evaluated the performance of the air bronchogram and BLL sign in differentiating GGNs, it did not provide a comprehensive evaluation of other morphological features that have been investigated in prior studies. Finally, given the low sensitivity of the BLL sign, it is recommended to combine it with other clinical and radiological features such as lobulation, spiculation, and pleural indentation signs to accurately assess the possibility of GGNs in clinical practice.

Reviewer #2:

**Q:** I did not understand how come the lesions ended up all to be resected.

**Reply:** Based on our observations, there are several reasons for the resection of nodules. Firstly, some patients have personal reasons such as anxiety or distress upon detecting the presence of a GGN on CT images. Regardless of the likelihood of the nodule being neoplastic or non-neoplastic, these patients strongly request surgical resection. Secondly, in cases where GGNs show stability or an increase in size or density during follow-up, there is a higher possibility of neoplastic lesions. In such instances, surgical resection is considered as a viable option. Thirdly, our hospital has achieved a high level of expertise in minimally invasive surgical techniques for removing pulmonary nodules. As a result, many patients opt for surgical removal as their preferred treatment option. These factors collectively contribute to the decision-making process for nodule resection at our hospital.

**Q:** Where there any tumor board discussion previously?

**Reply:** At present, tumor board discussion is only for a small number of patients in our hospital. The decision regarding the need for surgical intervention is typically made by the surgeon after considering the patient's individual circumstances and preferences through a collaborative discussion process.

**Q:** Where there any previously follow-up of the lesions?

**Reply:** In this study, a total of 287 patients had follow-up data available at our hospital. Among these patients, 70 cases were confirmed to have non-neoplastic lesions, while 217 cases were confirmed to have neoplastic lesions. Based on the follow-up CT data, it was observed that 51 GGNs increased in size, 14 increased in density, 9 showed an increase in both size and density, and 213 remained stable.

**Q:** What kind of surgery did you perform? Lobectomy? wedge resection?

**Reply:** In this study, 442 (24.3%) cases underwent wedge resection, 836 (46.1%) cases underwent segmentectomy, and 537 (29.6%) cases underwent lobectomy. These data have been included in Table 1.

**Q:** Histology was made onsite OR or delayed?

**Reply:** Both intraoperative frozen sections and paraffin sections were conducted for each lesion in this study. However, the final pathological result of lesions was based on the analysis of the paraffin sections.

**Q:** From the imaging analysis, there was not intravenous contrast? and lesions alone were not considered to have solid component

**Reply:** We determine the presence of solid components in GGNs by evaluating whether the lesion contains component that completely obscures the bronchial and vascular structures, rather than relying on enhancement results. In this study, all examinations included an unenhanced acquisition, and the examination also included additional contrast-enhanced images in some patients although the contrast-enhanced were not further evaluated as part of the present analysis.

**Changes in the text**:

The analysis was based on the unenhanced CT images, which were acquired with the following settings:.....

**Q:** Regarding demographics. Patients had any other comorbidities, namaly pulmonary or cardiac? Where smokers? where occupational lung diseases dismissed as well as other systemic conditions (namely auto-immune diseases) considered?

**Reply:** Based on the electronic medical record system and chest CT images, the patients’ concomitant diseases were collected and compared. We had added the statement and data in the “Methods” and table 1.

**Changes in the text**:

Clinical data and image analysis

Based on the electronic medical record system and chest CT images, the patients’ clinical data were recorded. Clinical data, including the patient’s age, sex, smoking history, and concomitant diseases (pneumonia, bronchiectasis, tuberculosis, interstitial pneumonia, autoimmune diseases, cardiac disease) were collected.

**Q:** How did you manage multiple GGN?

**Reply:** In this study, we identified a total of 20 patients who had multiple confirmed GGNs. Among these patients, 4 cases had multiple nodules located in the same segment, 7 cases had multiple nodules in the same lobe, and 9 cases had multiple nodules in different lobes. Importantly, all of these multiple nodules were successfully removed during a single surgery through video-assisted thoracoscopy.

Reviewer #3:

A. Patient selection.

**Q:** Patients with pulmonary emphysema were excluded because it makes the differentiation between emphysematous changes and GGL-related air containing spaces difficult. However, emphysema is a risk factor for pulmonary malignancy. Thus, potentially excluding a large number of patients.

**Reply:** In this study, a total of 28 patients with emphysema were excluded, including 8 cases with non-neoplastic GGNs and 20 cases with neoplastic ones. Among the 28 GGNs, only two neoplastic ones have BLL in them. Based on this data, we believe that the exclusion of patients with emphysema will not have a significant impact on the results of this study.

**Q:** The authors excluded lesions larger than 3 cm. The reason for this was not explained in the Methods section, and the reason for this selection is unclear.

**Reply:** According to the definition, pulmonary lesions larger than 3 cm are considered masses. In our study, we specifically focused on studying GGNs because surgically resected ones are predominantly observed as nodules rather than masses, particularly in non-neoplastic cases. As a result, we excluded lesions larger than 3 cm from our analysis. This exclusion criterion has been clearly annotated and explained in the flowchart of our study.

**Q:** It would be clarifying to know the extent of the patient selection. How many patients with GGN's and emphysema were excluded, and how many with GGNs larger than 3cm. This could be displayed in a diagram.

**Reply:** We've added a flowchart to the article. We conducted a retrospective analysis of a cohort of 3341 patients who underwent surgical resection for lung lesions between January 2015 and October 2022. Out of these patients, 1790 were included in the study based on the predefined criteria, while 1551 patients were excluded. The excluded 1551 patients included 31 (2.0%) with no preoperative chest CT ,19 (1.2%) with interval between preoperative chest CT and resection >1 month ,1375 (88.7%) cases with solid nodule or mass, 48 (3.1%) with CT images of thickness > 1mm, 33 (2.1%) with ground-glass lesions > 3 cm, 28 (1.8%) with emphysema, and 17 (1.1%) with artifacts on CT images or incomplete clinical data.

**Q:** Was the study population derived from referral after lung cancer screening or from referral to the thoracic surgeon?

**Reply:** In this study, a total of 1790 patients were included. Among them, 1203 (67.2%) patients were found to have nodules during routine screening for lung cancer. Additionally, 587 (32.8%) patients discovered pulmonary nodules incidentally during routine examinations for various other diseases, such as gastrointestinal conditions, cardiovascular issues, or emergency CT scans for chest pain.

B. Other indicators of malignancy of ground glass lesions

**Q:** Spiculation signs and pleural indentation sign were found to be significant correlators with malignancy in other studies (f.i. recent meta analysis) but had low AUC in the present study. Do the authors have an explanation for that?

**Reply:** We conducted an extensive literature review on the differentiation of GGNs. The significance of spiculation and pleural indentation signs in distinguishing between neoplastic and non-neoplastic lesions varied among the studies. In studies such as PMID: 34037864, PMID: 28822623, and PMID: 34993112, both signs were found to be significant in differentiation. However, in some studies like PMID: 32844603 and PMID: 30260240, neither spiculation sign nor pleural indentation sign showed significance in differentiation.

In our study, the spiculation and pleural indentation signs had lower AUC values compared to other studies. This difference may be attributed to variations in sample source, composition, and numbers across different studies. In our study, only surgically resected GGNs were included, whereas absorbable ones were also studied in some previous studies (PMID: 30260240, 34037864, 34993112). Moreover, in this study (PMID: 28822623), the composition ratio of benign and malignant nodules was 1:10. Additionally, the sample sizes in studies (PMID: 34037864 and PMID: 34993112) were relatively small (279 and 119 cases), which can introduce bias in the results.

Therefore, the significance of spiculation and pleural indentation signs in differentiating GGNs may vary in studies with different designs and characteristics.

C. Miscelaneous
Emphysema

**Q:** As the authors indicated, air-containing spaces in pGGN may be due to pre-existing emphysematous lesions. Emphysema was an exclusion criterium. Furthermore, emphysema is a risc factor for pulmonary malignancy and thus frequently co-exists with the presence of pulmonary malignancy and GGN's. Could the authors speculate on the consequence of this exclusion on their findings?

**Reply:** In this study, a total of 28 patients with emphysema were excluded, including 8 cases with non-neoplastic GGNs and 20 cases with neoplastic ones. Among the 28 GGNs, only two neoplastic ones have BLL in them. Based on this data, we believe that the exclusion of patients with emphysema will not have a significant impact on the results of this study.

Suggestion for conclusion of discussion (add underlined):

**Q:** In conclusion, by investigating the performance of air-containing space and its specific patterns (air bronchogram and BLL) in differentiating GGNs based on a larger sample in patients without emphysema, it was found that any one of them was more common in neoplastic GGNs and can be seen as predictive indicators.

**Reply:** Thank you very much for your suggestion. We have revised the sentence in the conclusion.

**Changes in the text:** In conclusion, by investigating the performance of air-containing space and its specific patterns (air bronchogram and BLL) in differentiating GGNs based on a larger sample in patients without emphysema……

Suggested recent papers for the reference list:
**Reply:** Thanks for your suggestion, we have added the papers that you suggested.

We hope all the corrections will meet with approval. If you have any questions, please contact me without any hesitation.

Sincerely yours,

Zhi-gang Chu

* Correspondent:

Zhi-gang Chu, M.D., Ph.D.

Email: chuzg0815@163.com

Department of Radiology

The First Affiliated Hospital of Chongqing Medical University

1# Youyi Road, Yuanjiagang, Yuzhong district

Chongqing

Tel.: +86 23 89012302

Fax: +86 23 68811487
